# Flexible and efficient recording of neuronal properties in large network simulations: The NEST Multimeter

**DOI:** 10.1186/1471-2202-12-S1-P92

**Published:** 2011-07-18

**Authors:** Hans E Plesser, Jochen M Eppler

**Affiliations:** 1 Department of Mathematical Sciences and Technology, Norwegian University of Life Sciences, 1432 Aas, Norway; 2 Institute of Neuroscience and Medicine, Computational and Systems Neuroscience (INM-6), Research Center Juelich, 52428 Juelich, Germany

## 

In computer simulations, all quantities are known and thus in principle available for recording and analysis. Collecting data from large simulations in an efficient and flexible manner is still challenging. Dumping all available data to file generates huge amounts of data, requiring significant post-processing to extract relevant information. Instrumenting all parts of a complex simulator for recording is also not trivial, especially with respect to file management and data collection in parallel simulations. We have developed the NEST Multimeter to facilitate flexible and efficient data recording from large-scale parallel neuronal network simulations with the NEST simulator [1]. Design goals were to: (i) allow users to selectively record neuron properties, e.g., membrane potential; (ii) provide a unified user interface for all types of recordings; (iii) support recording in parallel simulations; (iv) minimize the computation time overhead; and (v) provide an API that allows model developers to make any scalar quantity in a model neuron available for recording.

From NEST version 2.0.0-RC1 onward (available from http://www.nest-initiative.org), all NEST neuron models support multimeter recording and the user can easily enquire the recordable quantities:

reveals that the conductance-based adaptive exponential integrate-and-fire neuron model [2] supports recording of the membrane potential (V_m), excitatory and inhibitory synaptic conductances (g_ex, g_in) and the adaptation current (w). We record membrane potential and adaptation current by connecting a properly configured multimeter:

After simulating for 5ms, we obtain the recorded values from the multimeter:

Data can also be recorded to files, with one file per virtual process, ensuring conflict-free recording in parallel simulations. When data is retrieved as illustrated above, it is combined across threads, but not MPI processes. A multimeter can record from an arbitrary number of neurons.

Model developers instrument a model for recording by adding a small amount of boilerplate code to models: a UniversalDataLogger (UDL) member collecting recorded values temporarily, and a RecordablesMap (RM):

When the multimeter is connected to a neuron, it informs the UniversalDataLogger about which entries of the RM shall be recorded. Once per time step, the model neuron calls UDL::record_data(), which collects the required data using the access functions specified in the RM. Once per minimal delay interval, the multimeter collects the data from the UDLs of all connected neurons and stores it in memory or writes it to file.
